# FusionHub: A unified web platform for annotation and visualization of gene fusion events in human cancer

**DOI:** 10.1371/journal.pone.0196588

**Published:** 2018-05-01

**Authors:** Priyabrata Panigrahi, Abhay Jere, Krishanpal Anamika

**Affiliations:** LABS, Persistent Systems, Pingala-Aryabhata, Erandwane, Pune, India; University of Michigan, UNITED STATES

## Abstract

Gene fusion is a chromosomal rearrangement event which plays a significant role in cancer due to the oncogenic potential of the chimeric protein generated through fusions. At present many databases are available in public domain which provides detailed information about known gene fusion events and their functional role. Existing gene fusion detection tools, based on analysis of transcriptomics data usually report a large number of fusion genes as potential candidates, which could be either known or novel or false positives. Manual annotation of these putative genes is indeed time-consuming. We have developed a web platform FusionHub, which acts as integrated search engine interfacing various fusion gene databases and simplifies large scale annotation of fusion genes in a seamless way. In addition, FusionHub provides three ways of visualizing fusion events: circular view, domain architecture view and network view. Design of potential siRNA molecules through ensemble method is another utility integrated in FusionHub that could aid in siRNA-based targeted therapy. FusionHub is freely available at https://fusionhub.persistent.co.in.

## Introduction

**Fig 1:**
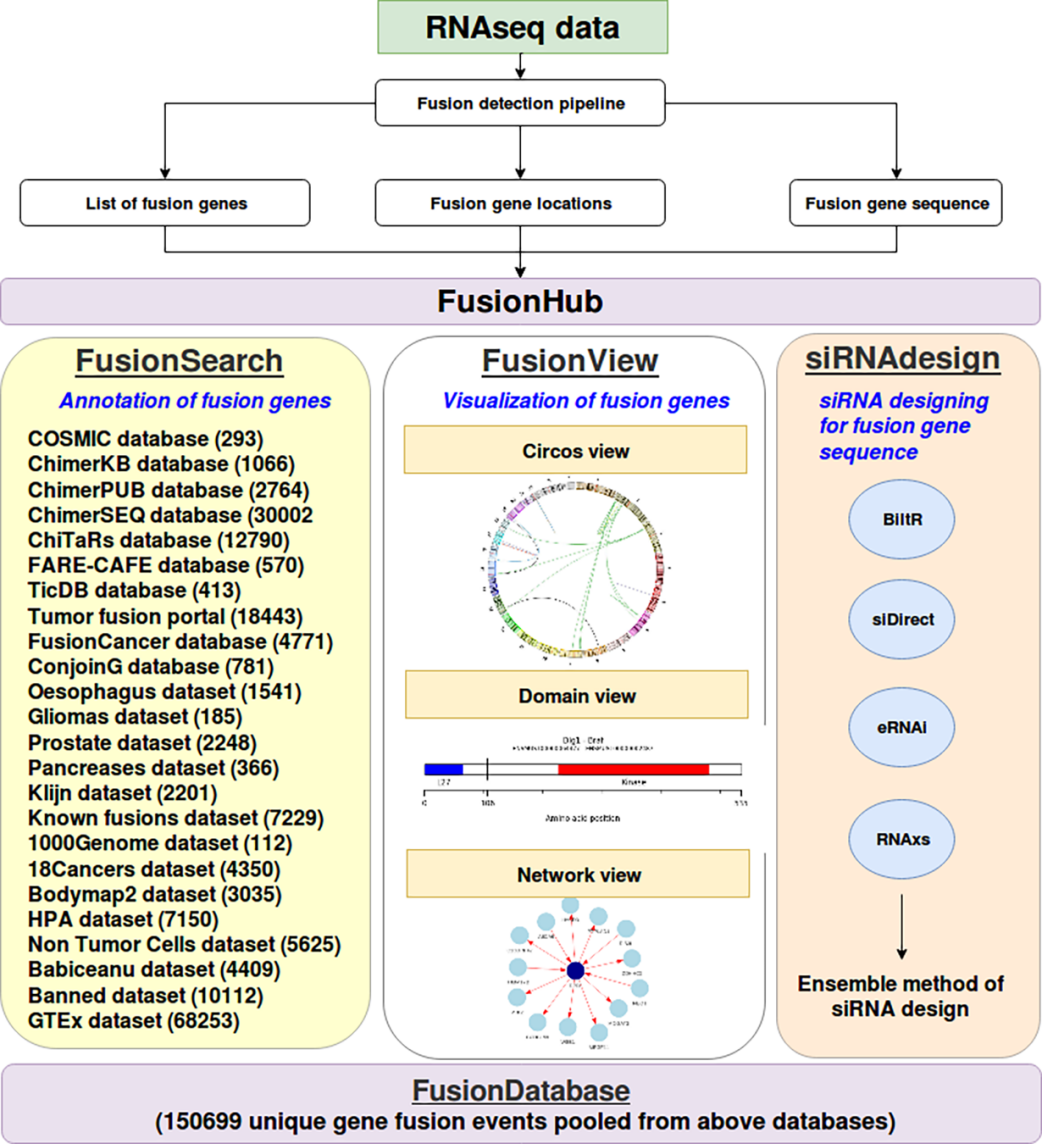
FusionHub server implementation with its three modules, FusionSearch, FusionView and siRNAdesign. The numbers in parenthesis in FusionSearch module correspond to number of fusion genes in that dataset. FusionDatabase consists of a total of 150699 fusion list, pooled from the 24 datasets listed in FusionSearch module.

Gene fusion is a chromosomal rearrangement event where two independent genes fuse together to form a hybrid gene. This rearrangement event usually involves insertion, deletion, inversion, translocation or read-through transcription of neighboring genes [1]. The chimeric protein thus produced as a result of fusion of genes often possesses oncogenic properties and such genes usually act as driver genes in cancer [1]. With the advent of Next Generation Sequencing (NGS) technology and development of powerful computational algorithms for gene fusion detection, rate of fusion detection has increased significantly [2]. Several databases have been developed that focus on information about fusion genes, their functional role, clinical association and inferred chromosomal breakpoints. Some of these databases are ChimerDB 3.0 (5 Jan 2018) (includes ChimerKB, ChimerSEQ and ChimerPUB) [3], TicDB Release 3.3 (2 Jan 2017) [4], COSMIC v83 (4 Jan 2018) [5], ChiTaRs Version 2.1 (2 Jan 2017) [6], FARE-CAFÉ (2 Jan 2017) [7], FusionCancer (3 Mar 2017) [8], Tumor Fusion Portal (4 Jan 2018) [9] and ConjoinG (3 Mar 2017) [10]. In addition to above databases, some other independent datasets are available in literature which provide list of validated fusion genes (Fig 1 and Table 1).

**Table 1:** Description of 24 fusion gene datasets included in FusionHub server.

Source	Database/Dataset	Total unique head—tail fusion gene pair	Dataset compiled from
*COSMIC v83 (4 Jan 2018)*	Database	293	COSMIC database [5]
*ChimerKB (ChimerDB 3)*		1066	ChimerKB database [3]
*ChimerPUB (ChimerDB 3)*		2764	ChimerPUB database [3]
*ChimerSEQ (ChimerDB 3)*		30002	ChimerSEQ database [3]
*ChiTaRs*		12790	ChiTaRs database [6]
*FARE-CAFE*		570	FARE-CAFE database [7]
*TicDB*		413	TicDB database [4]
*Tumor fusion portal*		18443	Tumor Fusion Gene Data Portal [9]
*FusionCancer*		4771	FusionCancer database [8]
*ConjoinG*		781	ConjoinG database [10]
*18Cancers*	Dataset	4350	Fusion genes observed in a RNA-seq dataset of 18 tumor types [11]
*Oesophagus*		1541	List of fusion genes seen in Oesophageal tumors from TCGA samples [12]
*Gliomas*		185	Fusion genes observed in RNA-seq dataset of glioblastoms [13]
*Prostate*		2248	Fusion genes observed in RNA-seq data from 150 prostate tumors [14]
*Pancreases*		366	Fusion genes observed in pancreatic tumor samples [15]
*Klijn*		2201	List of fusion genes from human cancer cell lines [16]
*Known_fusion*		7229	List of fusions known from literature. Compiled from FusionCatcher [17]
1000Genome		112	Fusion genes observed in samples from 1000genome project. Compiled from FusionCatcher [17]
Bodymap2		3035	List of fusion genes observed in healthy samples from 16 organs from Illumina Body Map RNAseq dataset. Compiled from FusionCatcher [17]
HPA		7150	Human Protein Atlas dataset; RNA-seq dataset of 27 healthy tissues [18]
Non_Tumor_Cells		5625	Fusions reported in non-tumor cell lines, compiled from FusionCatcher [17]
Babiceanu		4409	Fusion genes observed in non-cancer tissues and cells [19]
Banned		10112	List of fusion genes from healthy sample with strong supporting data. Compiled from FusionCatcher [17]

Each of the above mentioned databases though contain useful information about fusion genes, the information contained in them is highly heterogeneous. They differ with respect to their fusion gene entries, methodology of fusion detection, data sources and database size (Fig 1). A very poor overlap has been observed (S1 and S2 Figs) among these databases in terms of reported gene fusion lists, indicating a dire need for data integration. Hence, an interface which integrates all of the above segregated databases on a single platform where collated information about any fusion event can be analyzed would prove to be a useful tool in cancer research. Furthermore, currently available gene fusion detection tools based on analysis of transcriptomics data usually reports large number of gene fusions, thereby making analysis of these genes across multiple databases a time consuming and cumbersome process. Although every database listed above supports querying the database, searching multiple fusion genes in an automated manner i.e. batch annotation is currently not available. Presently no web tools or platforms exists which take list of fusion genes as input, search in all known fusion gene databases followed by an automated annotation process. In view of this, we present FusionSearch, a unique search engine where batch annotation of fusion genes can be carried out seamlessly.

In addition to annotation, visualization of gene fusion events is another important aspect for studying the underlying mechanism of gene fusion. We present FusionView, a comprehensive gene fusion visualization utility. It provides three different modes of fusion gene visualizations which are circular view, domain architecture view and network view. The circular view allows user to visualize various intra- and inter-chromosomal fusion events via a circular chromosome map. The domain architecture view depicts different ways by which head and tail genes fuse together via different combinations of exons and functional domains. The network view allows user to explore a fusion gene and its interaction partners through a dynamic fusion gene network. These views are capable of generating visualizations by either taking custom inputs from user or help them to visualize known fusion events from public domain databases.

The siRNA-based targeted therapy against fusion gene is emerging as one of the promising strategies for cancer treatment [20]. Currently several tools are available for siRNA designing but they differ in terms of their underlying algorithms. Since designing highly potent siRNA sequences relies completely on the underlying algorithm, we present an interface, siRNAdesign, that allows users to employ an ensemble of four siRNA prediction tools, namely, BiLTR [21], siDirect 2.0 [22], eRNAi 3.2 [23] and RNAxs [24], for siRNA designing.

The above three features, annotation (via FusionSearch module), visualization (via FusionView module) and siRNA designing (via siRNAdesign module) are integrated on a single platform *FusionHub*, making it a centralized hub for gene fusion data analysis. To the best of our knowledge, currently there is no web tool which provides these features on a single platform.

## Materials and methods

### Server designing and implementation

FusionHub is designed on a Linux platform using Perl, R, PHP and HTML. FusionHub provides three modules, namely FusionSearch, FusionView and siRNAdesign (Fig 1). For FusionSearch module, a local database (FusionDatabase) is created in the back end by searching for the keyword “gene fusion” and collecting fusion gene information from 10 public domain databases and 14 datasets from literature. We have included all the gene fusion entries present in these 24 databases. The sources and description of these 24 datasets are summarized in Table 1. The local database (FusionDatabase) is used at backend for obtaining fusion gene annotation. FusionDatabase is updated regularly in every three months. The web server is freely available at https://fusionhub.persistent.co.in.

### FusionSearch module

The FusionSearch module provides three options of searching: Gene pair-wise, Gene-wise and Chromosome-wise. In the Gene pair-wise search mode, fusion gene pair can be given as input, in the format of Headgene—Tailgene. For example, CCDC6—RET, where CCDC6 would be considered as head gene while RET as tail gene. On querying, the annotation for CCDC6—RET fusion gene would be retrieved. User can input one or more fusion gene pairs, one pair per line, allowing for batch annotation. An option is provided for users to search head or tail genes separately, in case where annotation for fusion gene pair is not found. In such cases all fusion events involving either head (e.g. CCDC6) or tail (e.g. RET) genes would be reported. In the Gene-wise search mode, one or more genes can be given as input, one per line (e.g. CCDC6). In this case, all fusion events involving the input gene (e.g. CCDC6) would be reported. In the Chromosome-wise search mode, users can retrieve information about all fusions between two chromosomes, both intra- and/or inter-chromosomal fusion events. An option is also provided to visualize gene fusion events in a form of a Circos [25] plot.

### FusionDatabase enrichment assessment

To assess the enrichment of currently available gene fusion databases with respect to their fusion list, we generated few test datasets from literature containing experimentally validated fusion genes. These test datasets were searched against fusion databases to check, for how many of these fusion genes, annotation is available. The search list included 27 fusion genes (Edgren_testset) from breast cancer dataset by Edgren, *et al*., 2011 [26], 11 fusion genes (Berger_testset) from melanoma dataset by Berger, *et al*., 2010 [27] and 9 genes (Yu_testset) from prostate cancer dataset by Yu, *et al*., 2014 [28]. These three datasets are widely used for benchmarking the performance of gene fusion detection tools [29, 30]. We have also included 113 clinically relevant recurrent fusion genes observed by Kumar-Sinha et al., 2015 [31] in epithelial cancers (Kumar_sinha_testset) to check how many of these clinically validated genes are enriched in databases. Further, we took a list of 2199 fusion genes reported by Klijn et al., 2015 (Klijn_testset), from 675 human cancer cell lines [16] and checked how many of these cell-line derived fusion genes are catalogued previously.

### The FusionView module

Circos [25] is used for visualization of gene fusion event in circular chromosome map while AGFusion [32] is used for obtaining domain architecture view. We use visNetwork and iGraph, R packages from CRAN (https://cran.r-project.org/), for visualization of gene fusion network. Each of these views support input of single or multiple fusion genes as input.

### The siRNAdesign module

The siRNAdesign module allows user to design potential siRNA molecules by selection of siRNA prediction tools of their choice. Currently it supports four widely used tools which are BiLTR [21], siDirect2.0 [22], eRNAi 3.2 [23] and RNAxs [24]. RNAxs and BiLTR are used locally while siDirect and eRNAi web server are used for siRNA prediction. Users can select one or more tool and tune tool specific parameters before submitting the job.

In every module, after successful completion of job, users are directed to the result page containing a unique job identification number which they can bookmark to retrieve the results later.

## Results and discussion

NGS based gene fusion detection involves several key analysis steps such as a) fusion detection, b) functional annotation, c) visualization, and d) validation. Several tools are available in public domain which aid in detection of fusion events using transcriptomics data [1]. These tools usually report large number of fusions of which many could be either known or novel or false positive. Therefore, it is important to annotate the detected fusion events by searching against known fusion databases. FusionHub acts as a unified platform for annotation of fusion genes by gathering information from many public domain databases (Fig 1). Visualization of fusion events and design of potential siRNA for any fusion sequence are other features available in FusionHub which provide users more insights into their data. In the following section, we will describe FusionSearch module followed by FusionView and siRNAdesign module, each with example and case studies.

### FusionSearch module

Our local database (FusionDatabase) consists of fusion genes compiled from a total of 24 datasets that include 10 publically available databases and 14 datasets from the literature (Table 1). Of the 14 datasets, some are derived from literature while others are compiled from FusionCatcher [17]. Datasets such as 1000genome, Bodymap2, HPA, Non_Tumor_Cells, Babiceanu, Banned and GTEx (Table 1) contain list of fusion genes observed in healthy samples. Therefore, a candidate fusion gene from a disease sample if found to be present in these datasets, might indicate a high probability of being false positive and therefore, should be analyzed carefully.

### FusionDatabase statistics

When fusion genes from all 24 datasets were pooled together, the total number of unique fusion events observed was 150699, involving a total of 25722 genes, of which 4297 act as head-only genes, 5312 act as tail-only gene while rest 16113 genes can act as head gene in some fusion and tail in other fusion events. When we compared the 24 datasets, each dataset was observed to differ with respect to their fusion gene list (Table 1, Fig 1) and a poor degree of overlap was observed among them (S1 and S2 Figs). A percent similarity matrix was generated to assess the degree of overlap among these databases. Percent similarity between any two datasets was measured as percent of common fusion genes; higher the value, higher is the similarity. Of the 24 datasets, only 2 dataset pair showed percent similarity values greater than 25; similarity between FARE-CAFE and TicDB was 70% while it was 60% between HPA and Banned dataset. For rest of the dataset pairs, similarity was observed to be less than 25% (S2 Fig), reflecting poor overlap.

**Fig 2:**
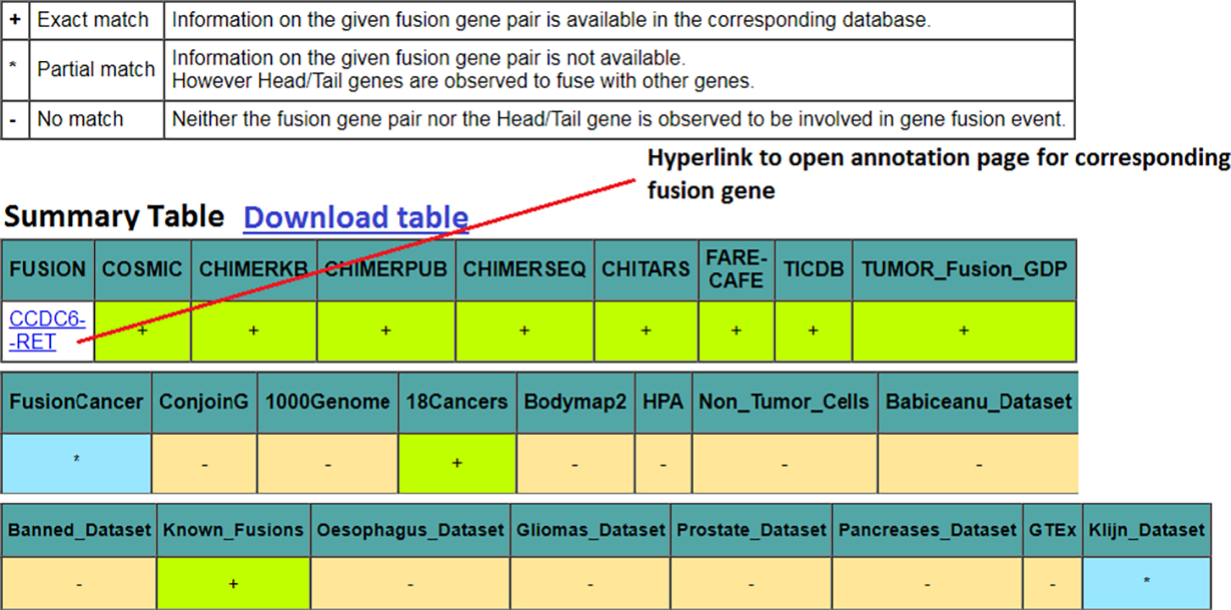
Summary page obtained after running FusionSearch module for CCDC6—RET gene. The result page can be browsed at https://fusionhub.persistent.co.in/cgi-bin/result_fetch_fusion.php?ID=Example1.

Fig 2 illustrates results for a sample input fusion gene (CCDC6—RET) submitted to FusionSearch module. A summary page is first displayed to the user that summarizes the distribution of fusion genes across all 24 databases. The plus sign (+) in the table represents exact match i.e. when the fusion gene was searched against a database, annotation was found to be present in the database for that gene. For example, CCDC6—RET gene fusion information is available in COSMIC, ChimerKB, ChimerPUB, ChimerSEQ, ChiTaRs, FARE-CAFE, TicDB, Tumorfusion portal databases along with 18Cancers and Known_fusion datasets. The star sign (*) represents partial match i.e. when fusion gene was searched, annotation for only one of the fused genes was found. For example, partial match is observed for CCDC6—RET fusion gene in FusionCancer and Klijn_Dataset, which means in FusionCancer and Klijn_Dataset, although no information for CCDC6—RET pair is available, CCDC6 and RET genes are individually observed to fuse with other genes. The minus sign (-) corresponds to no match i.e. neither exact match nor partial match (Fig 2).

**Fig 3:**
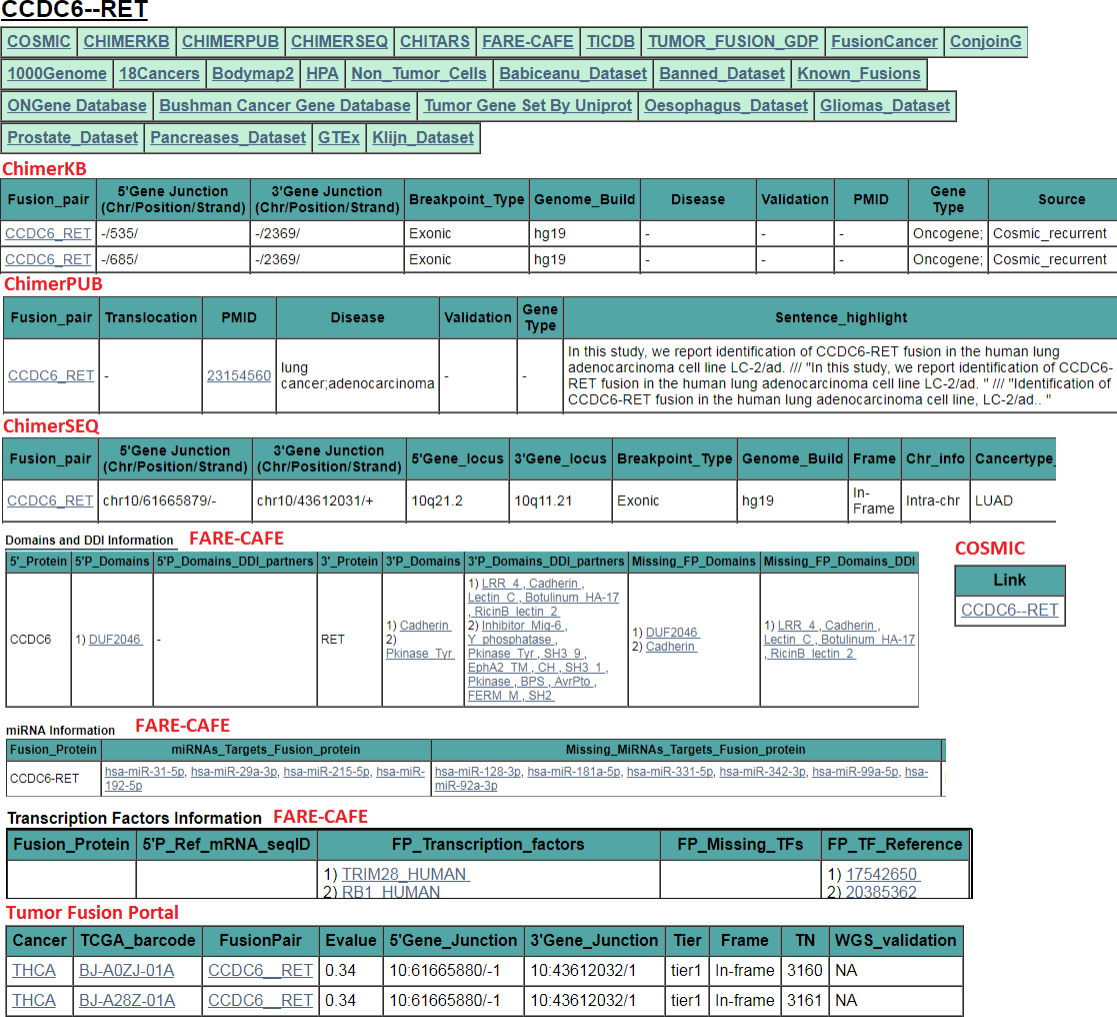
Annotations obtained for CCDC6—RET gene fusion from various databases. Only few annotations are shown here. Full annotation can be browsed at https://fusionhub.persistent.co.in/out/Example1/Individual/CCDC6—RET.html.

User can download the entire summary table as a tab delimited file. In the summary table, the first column displaying list of fusion genes is hyperlinked to the detailed annotation page. The annotation page allows users to glance through further detailed annotation of fusion gene such as its chromosomal breakpoints, disease details, protein-protein and domain-domain interactions of fusion genes, information about miRNAs and transcription factors regulating expression of fusion genes and other supporting experimental information. Sample annotation page for CCDC6—RET fusion gene is shown in Fig 3. Along with above annotations, FusionSearch also marks the head/tail genes as known oncogenes (if found in ONGene database [33]) or cancer associated (if found in Bushman cancer gene database, http://www.bushmanlab.org/links/genelists) or proto-oncogene or tumor suppressor (as per Uniprot database, http://www.uniprot.org/).

### FusionDatabase enrichment assessment

We further assessed the enrichment of FusionDatabase with respect to their fusion gene list by annotating five experimentally validated known fusion gene set and checked how often these are catalogued in these databases. The 5 test datasets consisted of a total of 2359 fusion genes from Edgren_testset (27 genes), Berger_testset (11 genes), Yu_testset (9 genes), Kumar-sinha_testset (113 genes) and Klijn_testset (2199 genes). Since COSMIC, ChimerKB, ChimerPUB and ChimerSEQ, ChiTaRs, FARE-CAFE, TicDB, Tumor fusion portal, FusionCancer and ConjoinG are most widely used and well curated databases compared to other 14 datasets, we are showing the enrichment results against these 10 databases only.

Table 2 shows the enrichment result. Of the 27 gene in Edgren_testset, annotation was observed for 19 genes in at least one database. ChimerDB3, ChiTaRs and Tumor fusion portal are the databases where annotation was observed for Edgren_testset genes. Similarly, of the 11 genes of Berger_testset and 9 genes of Yu_testset, only for 3 genes, annotation was found. While 75% (85 out of 113) of genes were annotated in case of Kumar_sinha_testset, the matching percentage was observed to be 16.57% in case of Klijn_testset. Since Klijn_testset consists of fusion genes observed in tumor-derived cell lines and majority of them (83.43%) have not been catalogued in public domain databases before, it opens the window for further investigation. For the test dataset considered, maximum enrichment was observed for ChimerSEQ database followed by Tumorfusion portal. Our study suggests need for more enrichment of existing databases to increase the sensitivity of fusion event detection.

**Table 2:** Enrichment of 10 databases included in FusionHub with respect to five test datasets obtained from literature. Detailed results can be browsed at https://fusionhub.persistent.co.in/fusionsearch_usecase_result.html.

	Edgren_testset	Berger_testset	Yu_testset	Kumar_sinha_testset	Klijn_testset	Total	Percentage match
Total genes in the test set	27	11	9	113	2199	2359	-
Total genes for which annotation found in at least one database	19	3	3	85	281	391	16.57
**Database**	**Database wise exact match count**					Total match count	Total match Percentage
COSMIC	0	0	2	45	20	67	2.84
CHIMERKB	0	0	2	68	32	102	4.32
CHIMERPUB	3	0	3	41	26	73	3.09
CHIMERSEQ	18	2	2	51	184	257	10.89
CHITARS	18	0	1	40	34	93	3.94
FARE.CAFE	0	0	2	53	19	74	3.14
TICDB	0	0	2	52	17	71	3.01
Tumor fusion portal	1	2	1	28	194	226	9.58
FusionCancer	0	0	2	7	15	24	1.02
ConjoinG	0	0	0	1	2	3	0.13

We believe that FusionDatabase, which at present consists of about 150699 compiled fusion genes, can be considered as a valuable resource for fusion gene annotation and assessing the performance of fusion detection tools.

### FusionView module

Visualization is an important aspect of data interpretation. In FusionHub server, we provide three different ways of fusion gene visualization a) Circular view b) Domain architecture view c) Fusion gene network view.

### Circular view

**Fig 4:**
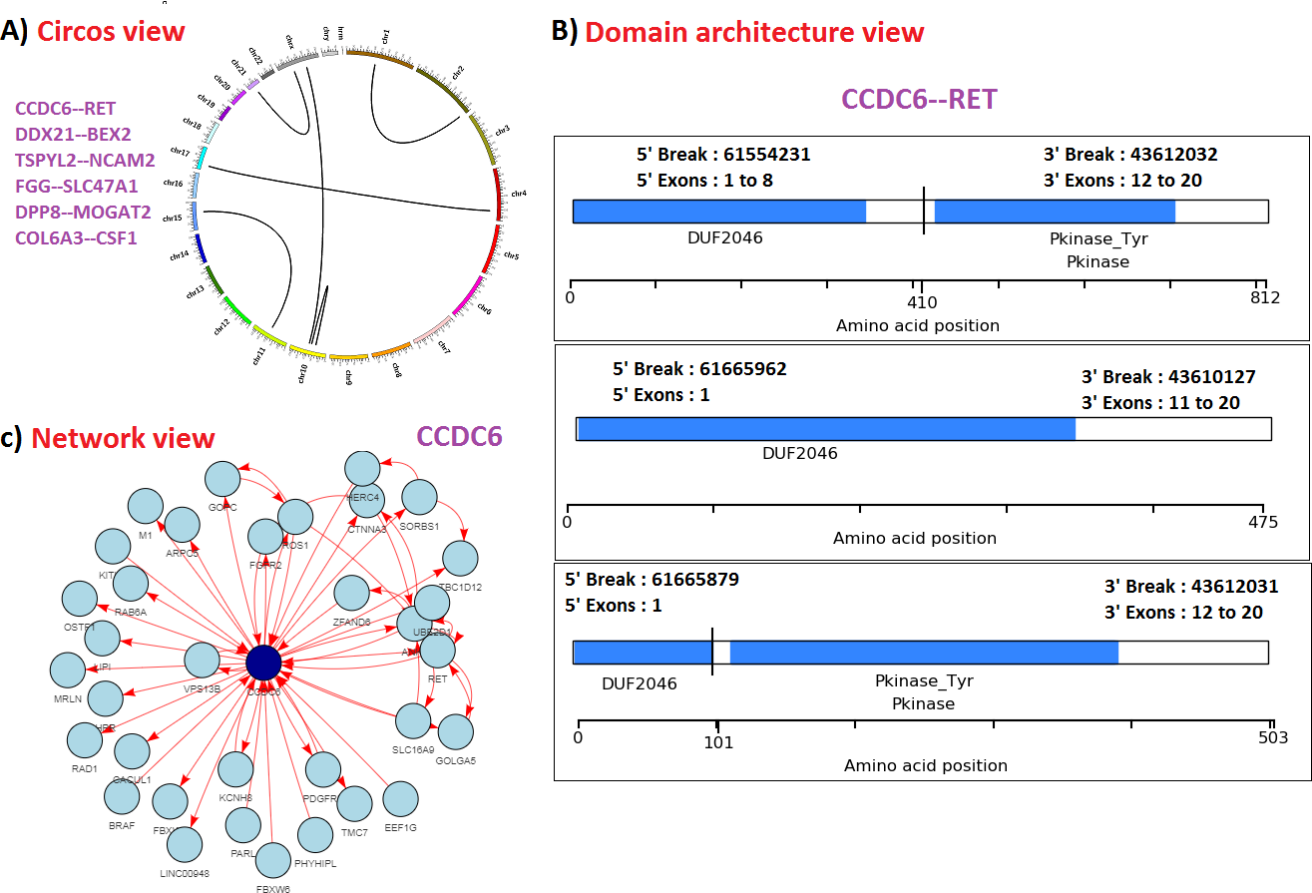
The different views available in FusionView module, circular view (A), domain architecture view (B) and network view (C). Circular view (A) shows fusion events for 6 input genes provided as input. Domain view shows three different ways by which CCDC6—RET fusion gene is formed (B). Network view shows the fusion gene network involving CCDC6 gene and its fusion interaction partners.

The circular representation of fusion events provides a holistic view of different chromosomes (inter and intra) involved in gene fusions (Fig 4A).

### Domain architecture view

Fusion translocation breakpoints in cancer are known to be non-random and mostly biased towards preservation of reading frame and functional viability of fusion protein [1]. Studies report that the rate of reading frame conservation is likely to be correlated with the rates of functional fusion genes. Breakpoints are observed to be selected such that splitting of functional domain can be avoided [1]. Studying different ways by which head/tail genes fuse together and maintain domain integrity is useful to understand the driving force behind gene fusion events. Currently no web platform is available which given a fusion gene, will display graphically all known fusion breakpoint locations, exonic structures of wild type head/tail gene and fusion genes, the protein domains of fusion genes. In view of this we have integrated domain architecture view feature in FusionView module. This utility is capable of analyzing large number of fusion genes as batch input. For a given fusion gene, all known breakpoint information is first fetched from FusionDatabase and then using AGFusion [32], the domain and exonic structures of wild type as well as fusion protein are displayed. The unique feature of this utility is to provide a comparative view wherein user can glance through the exon and domain combinations of fusion gene. Fig 4B shows three different ways by which CCDC6—RET fusion is formed. The three chimeric proteins differ with respect to protein length, the domain architecture and exons involved in fusion events.

### Fusion gene network view

Since the list of fusion genes are rapidly expanding with the advancement in technology, it is important to study genes that are frequently involved in fusion events and what partner they fuse with. Gene fusion network is a useful display to explore fusion partnerships. Currently no web platform exists, which, given an input gene will display all its known fusion partner and their interactions through a fusion gene network. In view of this we have integrated gene fusion network visualization in FusionView module. The module work in two ways, user can either input a single gene or provide a list of fusion genes. In the former case, all genes fused with the input genes are retrieved and fusion gene network is displayed. In the latter case, only the interactions involving the input fusion gene lists are displayed. Fig 4C shows gene fusion network involving CCDC6 gene. Network displayed are dynamic in nature with the features like node and edge selection, node filtering, nearest neighbor highlighting and network collapsing. Along with the visualization, this module further performs network analysis and identifies the most influential gene through degree centrality, closeness and betweenness.

### siRNA design module

siRNA-based targeted therapy against fusion gene is emerging as one of the promising strategies for cancer treatment [20]. siRNA designing depends mainly on the underlying algorithm and parameters of the tool. An ensemble approach of predictions by using multiple tools with different underlying algorithms can provide higher confidence in selecting a siRNA molecule if predicted by more than one tool. In view of this we have provided an interface where siRNA sequences can be designed using a combination of 4 prediction tools namely BiLTR [21], siDirect [22], eRNAi [23] and RNAxs [24]. The module first predicts siRNAs for fusion gene sequence by individual tools followed by a comparative report listing siRNA molecules predicted by more than one tools.

### siRNAdesign module case study

**Fig 5:**
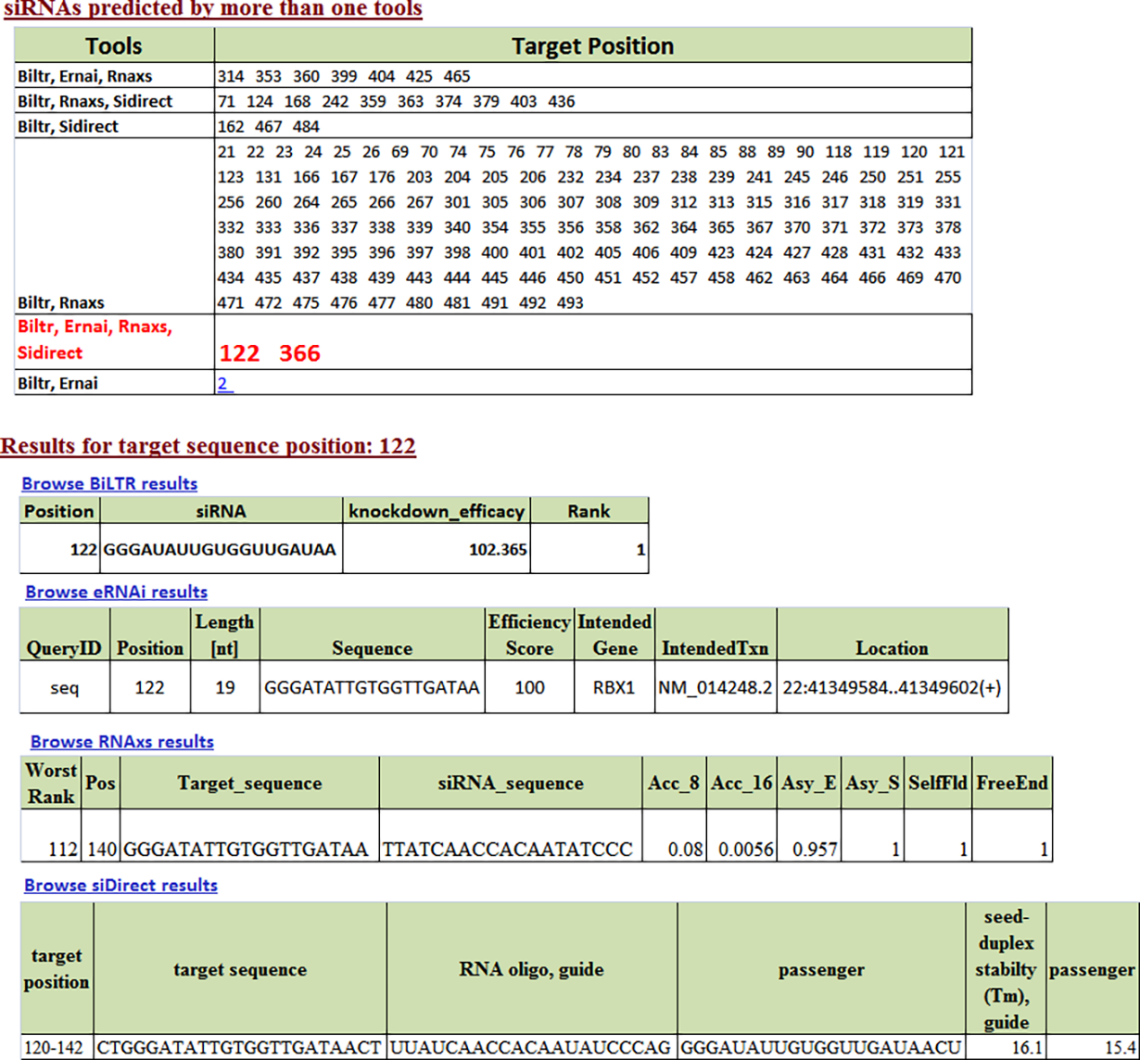
Result of siRNA prediction for RBX1 gene.

To demonstrate the usability of siRNAdesign ensemble approach, we selected 90 experimentally validated siRNAs present in MIT/ICBP siRNA database http://web.mit.edu/sirna/index.html (http://web.mit.edu/sirna/index.html) targeting 63 genes in human cancer. Each of these gene sequences were submitted to siRNAdesign module using all 4 siRNA prediction tools with default parameter settings. Of these 90 experimentally validated siRNAs, using the ensemble approach, 62 (68%) siRNAs could be correctly predicted by more than one tool (S1 Table). Of these, 4 siRNAs were predicted by all four tools while 12 siRNAs were predicted by at least three tools and the remaining 46 siRNAs were predicted by at least two tools. The ensemble approach would aid the user to confidently select potential siRNAs not only based on prediction scores of individual tools but also based on number of tools supporting the prediction. We show this by taking an example of siRNA prediction for RBX1 (ring-box 1) gene (Fig 5). This gene is considered as potential biomarkers of resistance to acyl sulfonamide-based cancer drugs. The siRNA designed at target position 122 is experimentally validated to be a potential siRNA candidate [34]. When siRNA was predicted for this gene, BiLTR, siDirect, eRNAi and RNAxs predicted a total of 503, 7, 10 and 154 possible siRNAs respectively. When ensemble approach is applied, the target position 122 was predicted to be potential siRNA by all the four tools. BiLTR predicted this siRNA as top-ranked siRNA with knock down efficiency score of 102.365 (Fig 5). siDirect also predicted this siRNA as the top ranked siRNA. While eRNAi predicted this siRNA as the top most siRNA with 100% knock down efficiency, RNAxs ranked this siRNA as 55^th^ from top. Based on ensemble approach, position 122 can be considered as potential siRNA as it is predicted by all four tools. Similar to position 122, ensemble approach also identified position 366 as potential siRNA, predicted by all the four tools (Fig 5). While eRNAi ranked this siRNA as 4^th^ from top with knockdown efficiency of 96.22%, siDirect ranked this siRNA 9^th^ from top. Similarly BiLTR ranked this siRNA as 62^nd^ from top with knock-down efficiency 84.51% and RNAxs ranked this 24^th^ from the top. One can thus apply filters based on tool specific prediction scores as well as number of tools supporting the prediction, to obtain highly potential siRNAs.

## Conclusions

In summary, we have developed FusionHub, a unified platform where annotation and different types of visualization of large number of gene fusion events is possible simultaneously along with siRNA designing for any given fusion gene sequence. With the advancement in high throughput sequencing technologies, it is certain that in near future fusion data would be generated at an exponential rate and therefore, availability of an integrated search engine interfacing several publicly available gene fusion repositories would be of great value for the scientific community.

## Supporting information

S1 Fig Heatmap showing percentage similarity among 24 datasets included in FusionHub. (DOCX)

S2 Fig A matrix showing percent similarity among 24 datasets included in FusionHub. (DOCX)

S1 Table Results of siRNA design module case study. Each row corresponds to one of the 90 siRNAs considered as test dataset. Columns from left to right correspond to target gene name, the MIT/ICBP siRNA Database ID for the siRNA, Target sequence for siRNA, experimentally measured mRNA knockdown efficiency, experimentally measured protein knockdown efficiency, target sequence position for siRNA, list of tools correctly predicted the corresponding siRNA, tool count, hyperlink to result and hyperlink to gene sequence. The row corresponding to gene RBX1 is described in the main text. The entire result can be browsed at http://fusionhub.persistent.co.in/sirna_usecase_result.html.
(DOCX)
