# Supplementary material for: FusionHub: A unified web platform for annotation and visualization of gene fusion events in human cancer
Source: PLoS One. 2018 May 1;13(5):e0196588. doi: 10.1371/journal.pone.0196588 (PMC5929557; doi:10.1371/journal.pone.0196588)
Supplement: S1 Fig — (DOCX) [file pone.0196588.s001.docx]

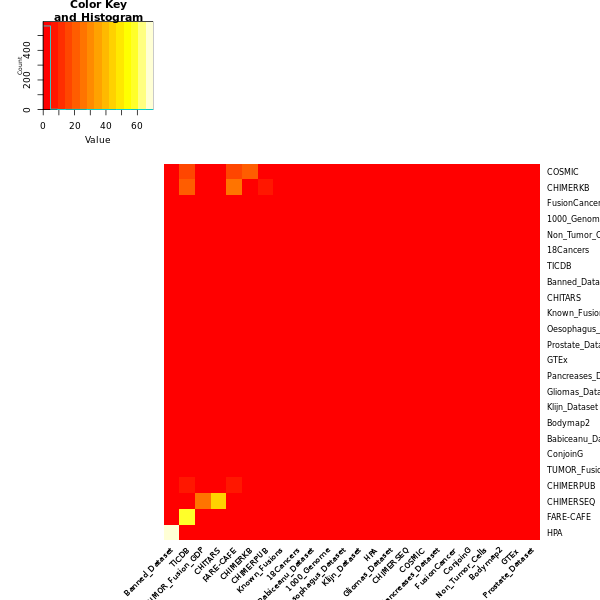


**S1 Figure:** Heatmap showing percentage similarity among the 24 datasets included in FusionHub. Percent similarity is a measure of degree of overlap between any two datasets. The heatmap scale is shown on top left corner with red indicating low percent similarity while yellow representing high percent similarity. Percent similarity between any two datasets is calculated as shown below

A = intersect (Dataset1 Fusion genes, Dataset2 Fusion genes)

B = union (Dataset1 Fusion genes, Dataset2 Fusion genes)

Percent similarity = (A/B)*100

It can be seen that most datasets are showing less degree of overlap with percent similarity value less than 25%. The yellow patches show those datasets which are having higher degree of overlap.
