# Supplementary material for: FusionHub: A unified web platform for annotation and visualization of gene fusion events in human cancer
Source: PLoS One. 2018 May 1;13(5):e0196588. doi: 10.1371/journal.pone.0196588 (PMC5929557; doi:10.1371/journal.pone.0196588)
Supplement: S2 Fig — (DOCX) [file pone.0196588.s002.docx]

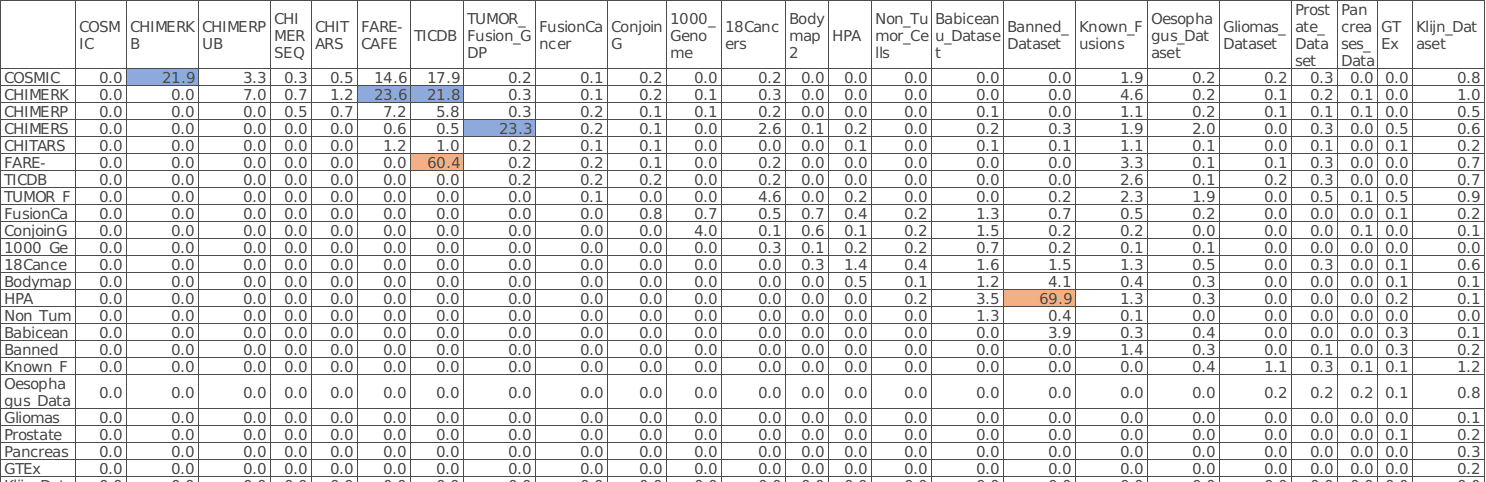


**S2 Figure:** Table showing percent similarity among 24 datasets which are included in FusionHub. Percent similarity values between 20 to 25% are highlighted in blue while those greater than 25 are marked in brown. Only two dataset pairs show similarity >25%.
