# Supplementary material for: FusionHub: A unified web platform for annotation and visualization of gene fusion events in human cancer
Source: PLoS One. 2018 May 1;13(5):e0196588. doi: 10.1371/journal.pone.0196588 (PMC5929557; doi:10.1371/journal.pone.0196588)
Supplement: S1 Table — Each row corresponds to one of the 90 siRNAs considered as test dataset. Columns from left to right correspond to target gene name, the MIT/ICBP siRNA Database ID for the siRNA, Target sequence for siRNA, experimentally measured mRNA knockdown efficiency, experimentally measured protein knockdown efficiency, target sequence position for siRNA, list of tools correctly predicted the corresponding siRNA, tool count, hyperlink to result and hyperlink to gene sequence. The row corresponding to gene RBX1 is described in the main text. The entire result can be browsed at http://fusionhub.persistent.co.in/sirna_usecase_result.html. (DOCX) [file pone.0196588.s003.docx]

**S1 Table:** Table displaying results of siRNA design module case study. Each row corresponds to one of the 90 siRNAs considered as test dataset. Columns from left to right correspond to target gene name, the MIT/ICBP siRNA Database ID for the siRNA, Target sequence for siRNA, experimentally measured mRNA knockdown efficiency, experimentally measured protein knockdown efficiency, target sequence position for siRNA, list of tools correctly predicted the corresponding siRNA, tool count, hyperlink to the result and hyperlink to gene sequence. The row corresponding to gene RBX1 is described in the main text. The entire result can be browsed at <http://fusionhub.persistent.co.in/sirna_usecase_result.html>

| **Target_Gene** | **siRNA_ID** | **Target_seq_for_sirna** | **mRNA knockdown** | **Protein knockdown** | **Target pos** | **Predicted by** | **Tool count** | **Prediction Result** | **Target sequence** |
| --- | --- | --- | --- | --- | --- | --- | --- | --- | --- |
| FH | [1063](http://web.mit.edu/sirna/sequences/results-1063.html) | GATCTACGATGAACTTTAA | 80-90% | --- | 253 | Biltr, Ernai, Rnaxs, Sidirect | 4 | [Case_1063](https://fusionhub.persistent.co.in/out/Case_1063/combinedresult.html#Lab253) | [View](https://fusionhub.persistent.co.in/out/Case_1063/sequence.html) |
| PLK1 | [1032](http://web.mit.edu/sirna/sequences/results-1032.html) | GGATCAAGAAGAATGAATA | 80-90% | --- | 838 | Biltr, Ernai, Rnaxs, Sidirect | 4 | [Case_1032](https://fusionhub.persistent.co.in/out/Case_1032/combinedresult.html#Lab838) | [View](https://fusionhub.persistent.co.in/out/Case_1032/sequence.html) |
| RBX1 | [1054](http://web.mit.edu/sirna/sequences/results-1054.html) | GGGATATTGTGGTTGATAA | 80-90% | --- | 122 | Biltr, Ernai, Rnaxs, Sidirect | 4 | [Case_1054](https://fusionhub.persistent.co.in/out/Case_1054/combinedresult.html#Lab122) | [View](https://fusionhub.persistent.co.in/out/Case_1054/sequence.html) |
| SFN | [1087](http://web.mit.edu/sirna/sequences/results-1087.html) | CATGTTTCCTCTCAATAAA | 70-80% | --- | 1280 | Biltr, Ernai, Rnaxs, Sidirect | 4 | [Case_1087](https://fusionhub.persistent.co.in/out/Case_1087/combinedresult.html#Lab1280) | [View](https://fusionhub.persistent.co.in/out/Case_1087/sequence.html) |
| YWHAZ | [1022](http://web.mit.edu/sirna/sequences/results-1022.html) | GGTTTATGTTACTTCTATT | 90-100% | 70-80% | 925 | Biltr, Rnaxs, Sidirect | 3 | [Case_1022](https://fusionhub.persistent.co.in/out/Case_1022/combinedresult.html#Lab925) | [View](https://fusionhub.persistent.co.in/out/Case_1022/sequence.html) |
| ASNS | [1060](http://web.mit.edu/sirna/sequences/results-1060.html) | GGATACTGCCAATAAGAAA | 80-90% | --- | 558 | Biltr, Rnaxs, Sidirect | 3 | [Case_1060](https://fusionhub.persistent.co.in/out/Case_1060/combinedresult.html#Lab558) | [View](https://fusionhub.persistent.co.in/out/Case_1060/sequence.html) |
| BRMS1 | [1051](http://web.mit.edu/sirna/sequences/results-1051.html) | GGAATAAGTACGAATGTGA | 80-90% | --- | 539 | Biltr, Rnaxs, Sidirect | 3 | [Case_1051](https://fusionhub.persistent.co.in/out/Case_1051/combinedresult.html#Lab539) | [View](https://fusionhub.persistent.co.in/out/Case_1051/sequence.html) |
| CAV1 | [1059](http://web.mit.edu/sirna/sequences/results-1059.html) | GCAACAATTTATGAATTGA | 80-90% | --- | 902 | Biltr, Rnaxs, Sidirect | 3 | [Case_1059](https://fusionhub.persistent.co.in/out/Case_1059/combinedresult.html#Lab902) | [View](https://fusionhub.persistent.co.in/out/Case_1059/sequence.html) |
| FH | [1039](http://web.mit.edu/sirna/sequences/results-1039.html) | CAACGATCATGTTAATAAA | 80-90% | --- | 563 | Biltr, Ernai, Rnaxs | 3 | [Case_1039](https://fusionhub.persistent.co.in/out/Case_1039/combinedresult.html#Lab563) | [View](https://fusionhub.persistent.co.in/out/Case_1039/sequence.html) |
| HPRT1 | [1045](http://web.mit.edu/sirna/sequences/results-1045.html) | GATTGTTGTTTAACTTGTA | 80-90% | --- | 961 | Biltr, Rnaxs, Sidirect | 3 | [Case_1045](https://fusionhub.persistent.co.in/out/Case_1045/combinedresult.html#Lab961) | [View](https://fusionhub.persistent.co.in/out/Case_1045/sequence.html) |
| KISS1 | [1041](http://web.mit.edu/sirna/sequences/results-1041.html) | GAAATGTTGCGTAACTCAA | 80-90% | --- | 697 | Biltr, Rnaxs, Sidirect | 3 | [Case_1041](https://fusionhub.persistent.co.in/out/Case_1041/combinedresult.html#Lab697) | [View](https://fusionhub.persistent.co.in/out/Case_1041/sequence.html) |
| ASNS | [1082](http://web.mit.edu/sirna/sequences/results-1082.html) | GAAGCTAAAGGTCTTGTTA | 70-80% | --- | 658 | Biltr, Rnaxs, Sidirect | 3 | [Case_1082](https://fusionhub.persistent.co.in/out/Case_1082/combinedresult.html#Lab658) | [View](https://fusionhub.persistent.co.in/out/Case_1082/sequence.html) |
| CCND1 | [1079](http://web.mit.edu/sirna/sequences/results-1079.html) | CCTACGATACGCTACTATA | 70-80% | --- | 1910 | Biltr, Rnaxs, Sidirect | 3 | [Case_1079](https://fusionhub.persistent.co.in/out/Case_1079/combinedresult.html#Lab1910) | [View](https://fusionhub.persistent.co.in/out/Case_1079/sequence.html) |
| K-RAS | [1105](http://web.mit.edu/sirna/sequences/results-1105.html) | GACGTATATTGTATCATTT | 70-80% | --- | 4218 | Biltr, Rnaxs, Sidirect | 3 | [Case_1105](https://fusionhub.persistent.co.in/out/Case_1105/combinedresult.html#Lab4218) | [View](https://fusionhub.persistent.co.in/out/Case_1105/sequence.html) |
| MAP2K1 | [1088](http://web.mit.edu/sirna/sequences/results-1088.html) | GGATCAAGTCCTGAAGAAA | 70-80% | 60-70% | 928 | Biltr, Rnaxs, Sidirect | 3 | [Case_1088](https://fusionhub.persistent.co.in/out/Case_1088/combinedresult.html#Lab928) | [View](https://fusionhub.persistent.co.in/out/Case_1088/sequence.html) |
| TOP1 | [1094](http://web.mit.edu/sirna/sequences/results-1094.html) | GGATGATGCTGATTATAAA | 70-80% | --- | 676 | Biltr, Ernai, Rnaxs | 3 | [Case_1094](https://fusionhub.persistent.co.in/out/Case_1094/combinedresult.html#Lab676) | [View](https://fusionhub.persistent.co.in/out/Case_1094/sequence.html) |
| ARHGDIB | [1026](http://web.mit.edu/sirna/sequences/results-1026.html) | CAGCTGGGTCCCTCTTCAA | 90-100% | --- | 794 | Biltr, Rnaxs | 2 | [Case_1026](https://fusionhub.persistent.co.in/out/Case_1026/combinedresult.html#Lab794) | [View](https://fusionhub.persistent.co.in/out/Case_1026/sequence.html) |
| CD82/KAI1 | [1024](http://web.mit.edu/sirna/sequences/results-1024.html) | CAAGGGTGTGTATATTGTA | 90-100% | --- | 1354 | Biltr, Rnaxs | 2 | [Case_1024](https://fusionhub.persistent.co.in/out/Case_1024/combinedresult.html#Lab1354) | [View](https://fusionhub.persistent.co.in/out/Case_1024/sequence.html) |
| HSP90A | [1025](http://web.mit.edu/sirna/sequences/results-1025.html) | AGAAATAGGTTAAACTGAA | 90-100% | --- | 2839 | Biltr, Rnaxs | 2 | [Case_1025](https://fusionhub.persistent.co.in/out/Case_1025/combinedresult.html#Lab2839) | [View](https://fusionhub.persistent.co.in/out/Case_1025/sequence.html) |
| RHOA | [1027](http://web.mit.edu/sirna/sequences/results-1027.html) | CGGAATGATGAGCACACAA | 90-100% | --- | 640 | Biltr, Rnaxs | 2 | [Case_1027](https://fusionhub.persistent.co.in/out/Case_1027/combinedresult.html#Lab640) | [View](https://fusionhub.persistent.co.in/out/Case_1027/sequence.html) |
| ABCB1 | [1053](http://web.mit.edu/sirna/sequences/results-1053.html) | CGAACACATTGGAAGGAAA | 80-90% | --- | 3501 | Biltr, Rnaxs | 2 | [Case_1053](https://fusionhub.persistent.co.in/out/Case_1053/combinedresult.html#Lab3501) | [View](https://fusionhub.persistent.co.in/out/Case_1053/sequence.html) |
| AKT2 | [1047](http://web.mit.edu/sirna/sequences/results-1047.html) | GGATGAAGTCGCTCACACA | 80-90% | --- | 872 | Biltr, Rnaxs | 2 | [Case_1047](https://fusionhub.persistent.co.in/out/Case_1047/combinedresult.html#Lab872) | [View](https://fusionhub.persistent.co.in/out/Case_1047/sequence.html) |
| ARD1A | [1049](http://web.mit.edu/sirna/sequences/results-1049.html) | AGATGAAATACTACTTCTA | 80-90% | --- | 191 | Biltr, Rnaxs | 2 | [Case_1049](https://fusionhub.persistent.co.in/out/Case_1049/combinedresult.html#Lab191) | [View](https://fusionhub.persistent.co.in/out/Case_1049/sequence.html) |
| ARHGDIB | [1066](http://web.mit.edu/sirna/sequences/results-1066.html) | CTGAATAAATAGATCAGAA | 80-90% | --- | 87 | Biltr, Rnaxs | 2 | [Case_1066](https://fusionhub.persistent.co.in/out/Case_1066/combinedresult.html#Lab87) | [View](https://fusionhub.persistent.co.in/out/Case_1066/sequence.html) |
| BAD | [1034](http://web.mit.edu/sirna/sequences/results-1034.html) | GAGTTTGTGGACTCCTTTA | 80-90% | --- | 599 | Biltr, Ernai | 2 | [Case_1034](https://fusionhub.persistent.co.in/out/Case_1034/combinedresult.html#Lab599) | [View](https://fusionhub.persistent.co.in/out/Case_1034/sequence.html) |
| BAG3 | [1052](http://web.mit.edu/sirna/sequences/results-1052.html) | GCAAAGAGGTGGATTCTAA | 80-90% | --- | 1334 | Biltr, Sidirect | 2 | [Case_1052](https://fusionhub.persistent.co.in/out/Case_1052/combinedresult.html#Lab1334) | [View](https://fusionhub.persistent.co.in/out/Case_1052/sequence.html) |
| BAG4 | [1065](http://web.mit.edu/sirna/sequences/results-1065.html) | GGATATAATCTGAGACAAA | 80-90% | --- | 2003 | Biltr, Rnaxs | 2 | [Case_1065](https://fusionhub.persistent.co.in/out/Case_1065/combinedresult.html#Lab2003) | [View](https://fusionhub.persistent.co.in/out/Case_1065/sequence.html) |
| CD44v6 | [1002](http://web.mit.edu/sirna/sequences/results-1002.html) | GCAACTCCTAGTAGTACAA | 80-90% | --- | 1337 | Biltr, Rnaxs | 2 | [Case_1002](https://fusionhub.persistent.co.in/out/Case_1002/combinedresult.html#Lab1337) | [View](https://fusionhub.persistent.co.in/out/Case_1002/sequence.html) |
| CTNNB1 | [1048](http://web.mit.edu/sirna/sequences/results-1048.html) | CGGGATGTTCACAACCGAA | 80-90% | 70-80% | 2012 | Biltr, Rnaxs | 2 | [Case_1048](https://fusionhub.persistent.co.in/out/Case_1048/combinedresult.html#Lab2012) | [View](https://fusionhub.persistent.co.in/out/Case_1048/sequence.html) |
| CTNNB1 | [1043](http://web.mit.edu/sirna/sequences/results-1043.html) | GGGTAGGGTAAATCAGTAA | 80-90% | 60-70% | 3557 | Biltr, Rnaxs | 2 | [Case_1043](https://fusionhub.persistent.co.in/out/Case_1043/combinedresult.html#Lab3557) | [View](https://fusionhub.persistent.co.in/out/Case_1043/sequence.html) |
| HSP90A | [1028](http://web.mit.edu/sirna/sequences/results-1028.html) | GCCCTAAGAGACAACTCAA | 80-90% | --- | 2042 | Biltr, Rnaxs | 2 | [Case_1028](https://fusionhub.persistent.co.in/out/Case_1028/combinedresult.html#Lab2042) | [View](https://fusionhub.persistent.co.in/out/Case_1028/sequence.html) |
| MMP1 | [1055](http://web.mit.edu/sirna/sequences/results-1055.html) | GCGTGTGACAGTAAGCTAA | 80-90% | --- | 900 | Biltr, Rnaxs | 2 | [Case_1055](https://fusionhub.persistent.co.in/out/Case_1055/combinedresult.html#Lab900) | [View](https://fusionhub.persistent.co.in/out/Case_1055/sequence.html) |
| NME2 | [1037](http://web.mit.edu/sirna/sequences/results-1037.html) | GAGCATATTTGCCAATAAA | 80-90% | --- | 789 | Biltr, Sidirect | 2 | [Case_1037](https://fusionhub.persistent.co.in/out/Case_1037/combinedresult.html#Lab789) | [View](https://fusionhub.persistent.co.in/out/Case_1037/sequence.html) |
| NME2 | [1033](http://web.mit.edu/sirna/sequences/results-1033.html) | GCACCTTCATCGCCATCAA | 80-90% | --- | 241 | Biltr, Rnaxs | 2 | [Case_1033](https://fusionhub.persistent.co.in/out/Case_1033/combinedresult.html#Lab241) | [View](https://fusionhub.persistent.co.in/out/Case_1033/sequence.html) |
| PLK1 | [1030](http://web.mit.edu/sirna/sequences/results-1030.html) | CCATATGAATTGTACAGAA | 80-90% | --- | 2085 | Biltr, Rnaxs | 2 | [Case_1030](https://fusionhub.persistent.co.in/out/Case_1030/combinedresult.html#Lab2085) | [View](https://fusionhub.persistent.co.in/out/Case_1030/sequence.html) |
| RHA | [1005](http://web.mit.edu/sirna/sequences/results-1005.html) | AGACGCAGAAGTTTTATGC | 80-90% | --- | 2574 | Biltr, Rnaxs | 2 | [Case_1005](https://fusionhub.persistent.co.in/out/Case_1005/combinedresult.html#Lab1) | [View](https://fusionhub.persistent.co.in/out/Case_1005/sequence.html) |
| RHOA | [1029](http://web.mit.edu/sirna/sequences/results-1029.html) | AGCTAGACGTGGGAAGAAA | 80-90% | --- | 816 | Biltr, Rnaxs | 2 | [Case_1029](https://fusionhub.persistent.co.in/out/Case_1029/combinedresult.html#Lab816) | [View](https://fusionhub.persistent.co.in/out/Case_1029/sequence.html) |
| ABCC1 | [1106](http://web.mit.edu/sirna/sequences/results-1106.html) | GAGTGGAATTCCGGAACTA | 70-80% | --- | 4050 | Biltr, Rnaxs | 2 | [Case_1106](https://fusionhub.persistent.co.in/out/Case_1106/combinedresult.html#Lab4050) | [View](https://fusionhub.persistent.co.in/out/Case_1106/sequence.html) |
| ADAM8 | [1085](http://web.mit.edu/sirna/sequences/results-1085.html) | GCATCATCGTCTACCGCAA | 70-80% | --- | 2107 | Biltr, Rnaxs | 2 | [Case_1085](https://fusionhub.persistent.co.in/out/Case_1085/combinedresult.html#Lab2107) | [View](https://fusionhub.persistent.co.in/out/Case_1085/sequence.html) |
| AKT3 | [1097](http://web.mit.edu/sirna/sequences/results-1097.html) | GCAGCTCCAACTTATATAA | 70-80% | --- | 2612 | Biltr, Rnaxs | 2 | [Case_1097](https://fusionhub.persistent.co.in/out/Case_1097/combinedresult.html#Lab2612) | [View](https://fusionhub.persistent.co.in/out/Case_1097/sequence.html) |
| ANP32A | [1103](http://web.mit.edu/sirna/sequences/results-1103.html) | GACTAAGTGGAATAACCTA | 70-80% | --- | 916 | Biltr, Sidirect | 2 | [Case_1103](https://fusionhub.persistent.co.in/out/Case_1103/combinedresult.html#Lab916) | [View](https://fusionhub.persistent.co.in/out/Case_1103/sequence.html) |
| ARHGEF12 | [1093](http://web.mit.edu/sirna/sequences/results-1093.html) | CGTTTAGCCCTGTCATTAA | 70-80% | --- | 3559 | Biltr, Sidirect | 2 | [Case_1093](https://fusionhub.persistent.co.in/out/Case_1093/combinedresult.html#Lab3559) | [View](https://fusionhub.persistent.co.in/out/Case_1093/sequence.html) |
| BAG1 | [1092](http://web.mit.edu/sirna/sequences/results-1092.html) | GGGTCATGTTAATTGGGAA | 70-80% | --- | 728 | Biltr, Rnaxs | 2 | [Case_1092](https://fusionhub.persistent.co.in/out/Case_1092/combinedresult.html#Lab728) | [View](https://fusionhub.persistent.co.in/out/Case_1092/sequence.html) |
| CAV1 | [1069](http://web.mit.edu/sirna/sequences/results-1069.html) | GGAATAAGTTCAAATTCTT | 70-80% | --- | 2121 | Biltr, Sidirect | 2 | [Case_1069](https://fusionhub.persistent.co.in/out/Case_1069/combinedresult.html#Lab2121) | [View](https://fusionhub.persistent.co.in/out/Case_1069/sequence.html) |
| CDK4 | [1078](http://web.mit.edu/sirna/sequences/results-1078.html) | GGTAATCCGGAGTGAGCAA | 70-80% | --- | 1190 | Biltr, Rnaxs | 2 | [Case_1078](https://fusionhub.persistent.co.in/out/Case_1078/combinedresult.html#Lab1190) | [View](https://fusionhub.persistent.co.in/out/Case_1078/sequence.html) |
| CUL2 | [1075](http://web.mit.edu/sirna/sequences/results-1075.html) | GCACAATGCCCTTATTCAA | 70-80% | --- | 2312 | Biltr, Sidirect | 2 | [Case_1075](https://fusionhub.persistent.co.in/out/Case_1075/combinedresult.html#Lab2312) | [View](https://fusionhub.persistent.co.in/out/Case_1075/sequence.html) |
| EGFR | [1084](http://web.mit.edu/sirna/sequences/results-1084.html) | GGATAGTATGAGCCCTAAA | 70-80% | 70-80% | 3898 | Biltr, Rnaxs | 2 | [Case_1084](https://fusionhub.persistent.co.in/out/Case_1084/combinedresult.html#Lab3898) | [View](https://fusionhub.persistent.co.in/out/Case_1084/sequence.html) |
| EGFR | [1091](http://web.mit.edu/sirna/sequences/results-1091.html) | CCTTAGCAGTCTTATCTAA | 70-80% | 80-90% | 610 | Biltr, Rnaxs | 2 | [Case_1091](https://fusionhub.persistent.co.in/out/Case_1091/combinedresult.html#Lab610) | [View](https://fusionhub.persistent.co.in/out/Case_1091/sequence.html) |
| GSK3B | [1086](http://web.mit.edu/sirna/sequences/results-1086.html) | CACTGGTCACGTTTGGAAA | 70-80% | 70-80% | 2347 | Biltr, Rnaxs | 2 | [Case_1086](https://fusionhub.persistent.co.in/out/Case_1086/combinedresult.html#Lab2347) | [View](https://fusionhub.persistent.co.in/out/Case_1086/sequence.html) |
| GSK3B | [1089](http://web.mit.edu/sirna/sequences/results-1089.html) | GCATTTATCGTTAACCTAA | 70-80% | 80-90% | 895 | Biltr, Sidirect | 2 | [Case_1089](https://fusionhub.persistent.co.in/out/Case_1089/combinedresult.html#Lab895) | [View](https://fusionhub.persistent.co.in/out/Case_1089/sequence.html) |
| HDAC1 | [1102](http://web.mit.edu/sirna/sequences/results-1102.html) | CCCGGAGGAAAGTCTGTTA | 70-80% | --- | 83 | Biltr, Rnaxs | 2 | [Case_1102](https://fusionhub.persistent.co.in/out/Case_1102/combinedresult.html#Lab83) | [View](https://fusionhub.persistent.co.in/out/Case_1102/sequence.html) |
| IGF2R | [1068](http://web.mit.edu/sirna/sequences/results-1068.html) | GAGTCTCGTACTATATAAA | 70-80% | --- | 6210 | Biltr, Rnaxs | 2 | [Case_1068](https://fusionhub.persistent.co.in/out/Case_1068/combinedresult.html#Lab6210) | [View](https://fusionhub.persistent.co.in/out/Case_1068/sequence.html) |
| MAP2K1 | [1073](http://web.mit.edu/sirna/sequences/results-1073.html) | GGAAGAATTCCTGAACAAA | 70-80% | 80-90% | 950 | Biltr, Sidirect | 2 | [Case_1073](https://fusionhub.persistent.co.in/out/Case_1073/combinedresult.html#Lab950) | [View](https://fusionhub.persistent.co.in/out/Case_1073/sequence.html) |
| MAPK14 | [1083](http://web.mit.edu/sirna/sequences/results-1083.html) | CTCTCCAAATATTATTCAA | 70-80% | --- | 1626 | Biltr, Rnaxs | 2 | [Case_1083](https://fusionhub.persistent.co.in/out/Case_1083/combinedresult.html#Lab1626) | [View](https://fusionhub.persistent.co.in/out/Case_1083/sequence.html) |
| NME1 | [1071](http://web.mit.edu/sirna/sequences/results-1071.html) | AGTTGGCAGGAACATTATA | 70-80% | --- | 441 | Biltr, Rnaxs | 2 | [Case_1071](https://fusionhub.persistent.co.in/out/Case_1071/combinedresult.html#Lab441) | [View](https://fusionhub.persistent.co.in/out/Case_1071/sequence.html) |
| RHOC | [1101](http://web.mit.edu/sirna/sequences/results-1101.html) | CTACTGTCTTTGAGAACTA | 70-80% | --- | 509 | Biltr, Rnaxs | 2 | [Case_1101](https://fusionhub.persistent.co.in/out/Case_1101/combinedresult.html#Lab509) | [View](https://fusionhub.persistent.co.in/out/Case_1101/sequence.html) |
| SFN | [1100](http://web.mit.edu/sirna/sequences/results-1100.html) | GGGAGAAGGTGGAGACTGA | 70-80% | --- | 325 | Biltr, Rnaxs | 2 | [Case_1100](https://fusionhub.persistent.co.in/out/Case_1100/combinedresult.html#Lab325) | [View](https://fusionhub.persistent.co.in/out/Case_1100/sequence.html) |
| SOS1 | [1062](http://web.mit.edu/sirna/sequences/results-1062.html) | GGTTGAATCCATCACTAAA | 70-80% | --- | 2228 | Biltr, Sidirect | 2 | [Case_1062](https://fusionhub.persistent.co.in/out/Case_1062/combinedresult.html#Lab2228) | [View](https://fusionhub.persistent.co.in/out/Case_1062/sequence.html) |
| SRC | [1104](http://web.mit.edu/sirna/sequences/results-1104.html) | GCTTGTGGGTGATGTTTGA | 70-80% | --- | 3029 | Biltr, Rnaxs | 2 | [Case_1104](https://fusionhub.persistent.co.in/out/Case_1104/combinedresult.html#Lab3029) | [View](https://fusionhub.persistent.co.in/out/Case_1104/sequence.html) |
| STAT1 | [1095](http://web.mit.edu/sirna/sequences/results-1095.html) | GCTTGACGTAGGAACGGTA | 70-80% | --- | 3040 | Biltr, Rnaxs | 2 | [Case_1095](https://fusionhub.persistent.co.in/out/Case_1095/combinedresult.html#Lab3040) | [View](https://fusionhub.persistent.co.in/out/Case_1095/sequence.html) |
| STAT3 | [1076](http://web.mit.edu/sirna/sequences/results-1076.html) | CGTCATTAGCAGAATCTCA | 70-80% | --- | 1021 | Biltr, Rnaxs | 2 | [Case_1076](https://fusionhub.persistent.co.in/out/Case_1076/combinedresult.html#Lab1021) | [View](https://fusionhub.persistent.co.in/out/Case_1076/sequence.html) |
| VIL2 | [1061](http://web.mit.edu/sirna/sequences/results-1061.html) | GGACTGATTGAATTACGGA | 70-80% | --- | 2232 | Biltr, Rnaxs | 2 | [Case_1061](https://fusionhub.persistent.co.in/out/Case_1061/combinedresult.html#Lab2232) | [View](https://fusionhub.persistent.co.in/out/Case_1061/sequence.html) |
| ITGB1 | [1023](http://web.mit.edu/sirna/sequences/results-1023.html) | GGATATTACTCAGATCCAA | 90-100% | --- | - | - | 0 | [Case_1023](https://fusionhub.persistent.co.in/out/Case_1023/combinedresult.html#Lab453) | [View](https://fusionhub.persistent.co.in/out/Case_1023/sequence.html) |
| XRN-1 beta | [1006](http://web.mit.edu/sirna/sequences/results-1006.html) | GTACCTGGATATACTAAGA | 90-100% | --- | - | - | 0 | [Case_1006](https://fusionhub.persistent.co.in/out/Case_1006/combinedresult.html) | [View](https://fusionhub.persistent.co.in/out/Case_1006/sequence.html) |
| XRN-1 delta | [1007](http://web.mit.edu/sirna/sequences/results-1007.html) | CUACUCAAGUACCUACUAA | 90-100% | --- | - | - | 0 | [Case_1007](https://fusionhub.persistent.co.in/out/Case_1007/combinedresult.html) | [View](https://fusionhub.persistent.co.in/out/Case_1007/sequence.html) |
| ARHGEF12 | [1067](http://web.mit.edu/sirna/sequences/results-1067.html) | GAGAGTCACCAACAGATAA | 80-90% | --- | 412 | - | 0 | [Case_1067](https://fusionhub.persistent.co.in/out/Case_1067/combinedresult.html#Lab412) | [View](https://fusionhub.persistent.co.in/out/Case_1067/sequence.html) |
| BAG3 | [1046](http://web.mit.edu/sirna/sequences/results-1046.html) | CGATGTGTGCTTTAGGGAA | 80-90% | --- | 2065 | - | 0 | [Case_1046](https://fusionhub.persistent.co.in/out/Case_1046/combinedresult.html#Lab2065) | [View](https://fusionhub.persistent.co.in/out/Case_1046/sequence.html) |
| BAG4 | [1044](http://web.mit.edu/sirna/sequences/results-1044.html) | ACATATACTTCATGTGTAA | 80-90% | --- | 2086 | - | 0 | [Case_1044](https://fusionhub.persistent.co.in/out/Case_1044/combinedresult.html#Lab2086) | [View](https://fusionhub.persistent.co.in/out/Case_1044/sequence.html) |
| CD82/KAI1 | [1031](http://web.mit.edu/sirna/sequences/results-1031.html) | GGGTTCTCTTATCAACTCA | 80-90% | --- | 0 | - | 0 | [Case_1031](https://fusionhub.persistent.co.in/out/Case_1031/combinedresult.html#Lab0) | [View](https://fusionhub.persistent.co.in/out/Case_1031/sequence.html) |
| CDH1 | [1050](http://web.mit.edu/sirna/sequences/results-1050.html) | GGCCTGAAGTGACTCGTAA | 80-90% | 60-70% | 2460 | - | 0 | [Case_1050](https://fusionhub.persistent.co.in/out/Case_1050/combinedresult.html#Lab2460) | [View](https://fusionhub.persistent.co.in/out/Case_1050/sequence.html) |
| FGFR1 | [1042](http://web.mit.edu/sirna/sequences/results-1042.html) | GAGATTTACCCATCGGGTA | 80-90% | --- | 3844 | - | 0 | [Case_1042](https://fusionhub.persistent.co.in/out/Case_1042/combinedresult.html#Lab3844) | [View](https://fusionhub.persistent.co.in/out/Case_1042/sequence.html) |
| GIPC1 | [1058](http://web.mit.edu/sirna/sequences/results-1058.html) | GCCGTACCTTCACGCTGAA | 80-90% | --- | 0 | - | 0 | [Case_1058](https://fusionhub.persistent.co.in/out/Case_1058/combinedresult.html#Lab0) | [View](https://fusionhub.persistent.co.in/out/Case_1058/sequence.html) |
| HIF1A | [1057](http://web.mit.edu/sirna/sequences/results-1057.html) | CGTTGTGAGTGGTATTATT | 80-90% | --- | 1304 | - | 0 | [Case_1057](https://fusionhub.persistent.co.in/out/Case_1057/combinedresult.html#Lab1304) | [View](https://fusionhub.persistent.co.in/out/Case_1057/sequence.html) |
| IGF2R | [1038](http://web.mit.edu/sirna/sequences/results-1038.html) | CGCAGGTAACGATGGGAAA | 80-90% | --- | 1459 | - | 0 | [Case_1038](https://fusionhub.persistent.co.in/out/Case_1038/combinedresult.html#Lab1459) | [View](https://fusionhub.persistent.co.in/out/Case_1038/sequence.html) |
| ITGB1 | [1056](http://web.mit.edu/sirna/sequences/results-1056.html) | GCGAGTGTGATAATTTCAA | 80-90% | --- | 1784 | - | 0 | [Case_1056](https://fusionhub.persistent.co.in/out/Case_1056/combinedresult.html#Lab1784) | [View](https://fusionhub.persistent.co.in/out/Case_1056/sequence.html) |
| PIK3C2A | [1036](http://web.mit.edu/sirna/sequences/results-1036.html) | CCCACTAATTGCATTGGAA | 80-90% | --- | 2880 | - | 0 | [Case_1036](https://fusionhub.persistent.co.in/out/Case_1036/combinedresult.html#Lab2880) | [View](https://fusionhub.persistent.co.in/out/Case_1036/sequence.html) |
| RB1 | [1035](http://web.mit.edu/sirna/sequences/results-1035.html) | CGTGTAAATTCTACTGCAA | 80-90% | 70-80% | 2027 | - | 0 | [Case_1035](https://fusionhub.persistent.co.in/out/Case_1035/combinedresult.html#Lab2027) | [View](https://fusionhub.persistent.co.in/out/Case_1035/sequence.html) |
| RB1 | [1040](http://web.mit.edu/sirna/sequences/results-1040.html) | GGGTTGTGTCGAAATTGGA | 80-90% | 70-80% | 1474 | - | 0 | [Case_1040](https://fusionhub.persistent.co.in/out/Case_1040/combinedresult.html#Lab1474) | [View](https://fusionhub.persistent.co.in/out/Case_1040/sequence.html) |
| AKT3 | [1098](http://web.mit.edu/sirna/sequences/results-1098.html) | GAGTGATCATGGAAATGTA | 70-80% | --- | 3313 | - | 0 | [Case_1098](https://fusionhub.persistent.co.in/out/Case_1098/combinedresult.html#Lab3313) | [View](https://fusionhub.persistent.co.in/out/Case_1098/sequence.html) |
| BRMS1 | [1064](http://web.mit.edu/sirna/sequences/results-1064.html) | GGAGAAGTTGTTCAGGGAA | 70-80% | --- | 372 | - | 0 | [Case_1064](https://fusionhub.persistent.co.in/out/Case_1064/combinedresult.html#Lab372) | [View](https://fusionhub.persistent.co.in/out/Case_1064/sequence.html) |
| CDK4 | [1081](http://web.mit.edu/sirna/sequences/results-1081.html) | GAACTGACCGGGAGATCAA | 70-80% | --- | 537 | - | 0 | [Case_1081](https://fusionhub.persistent.co.in/out/Case_1081/combinedresult.html#Lab537) | [View](https://fusionhub.persistent.co.in/out/Case_1081/sequence.html) |
| FMR1 | [1074](http://web.mit.edu/sirna/sequences/results-1074.html) | CGTGAATGGAGTACCCTAA | 70-80% | --- | 2110 | - | 0 | [Case_1074](https://fusionhub.persistent.co.in/out/Case_1074/combinedresult.html#Lab2110) | [View](https://fusionhub.persistent.co.in/out/Case_1074/sequence.html) |
| GIPC1 | [1090](http://web.mit.edu/sirna/sequences/results-1090.html) | ACATGATCGAGGCCATTAA | 70-80% | --- | 0 | - | 0 | [Case_1090](https://fusionhub.persistent.co.in/out/Case_1090/combinedresult.html#Lab0) | [View](https://fusionhub.persistent.co.in/out/Case_1090/sequence.html) |
| K-RAS | [1096](http://web.mit.edu/sirna/sequences/results-1096.html) | GAGATAACACGATGCGTAT | 70-80% | --- | 3460 | - | 0 | [Case_1096](https://fusionhub.persistent.co.in/out/Case_1096/combinedresult.html#Lab3460) | [View](https://fusionhub.persistent.co.in/out/Case_1096/sequence.html) |
| MET | [1072](http://web.mit.edu/sirna/sequences/results-1072.html) | CGAGGGAATCATCATGAAA | 70-80% | 90-100% | 3580 | - | 0 | [Case_1072](https://fusionhub.persistent.co.in/out/Case_1072/combinedresult.html#Lab3580) | [View](https://fusionhub.persistent.co.in/out/Case_1072/sequence.html) |
| P53 | [1099](http://web.mit.edu/sirna/sequences/results-1099.html) | GAGTGCATTGTGAGGGTTA | 70-80% | 80-90% | 1804 | - | 0 | [Case_1099](https://fusionhub.persistent.co.in/out/Case_1099/combinedresult.html#Lab1804) | [View](https://fusionhub.persistent.co.in/out/Case_1099/sequence.html) |
| SMAD2 | [1070](http://web.mit.edu/sirna/sequences/results-1070.html) | ACTAGAATGTGCACCATAA | 70-80% | --- | 1625 | - | 0 | [Case_1070](https://fusionhub.persistent.co.in/out/Case_1070/combinedresult.html#Lab1625) | [View](https://fusionhub.persistent.co.in/out/Case_1070/sequence.html) |
| STAT1 | [1077](http://web.mit.edu/sirna/sequences/results-1077.html) | CAACTATATTATCATGCAA | 70-80% | --- | 3937 | - | 0 | [Case_1077](https://fusionhub.persistent.co.in/out/Case_1077/combinedresult.html#Lab3937) | [View](https://fusionhub.persistent.co.in/out/Case_1077/sequence.html) |
| TOP1 | [1080](http://web.mit.edu/sirna/sequences/results-1080.html) | GGAAGGACTCCATCAGATA | 70-80% | --- | 1842 | - | 0 | [Case_1080](https://fusionhub.persistent.co.in/out/Case_1080/combinedresult.html#Lab1842) | [View](https://fusionhub.persistent.co.in/out/Case_1080/sequence.html) |
| CXCR4 | [1000](http://web.mit.edu/sirna/sequences/results-1000.html) | GTTTTCACTCCAGCTAACA | --- | 90-100% | 0 | - | 0 | [Case_1000](https://fusionhub.persistent.co.in/out/Case_1000/combinedresult.html#Lab0) | [View](https://fusionhub.persistent.co.in/out/Case_1000/sequence.html) |
